# Paraphimosis

**Published:** 1876-10

**Authors:** J. C. Watson

**Affiliations:** Saltville, Va.


### PARAPHIMOSIS.

BY J. C. WATSON, M. D., SALTVILLE, VA.

*To the Editors of the Southern Medical Record:*

I propose to mention briefly the very simple plan of relieving the very troublesome little accident (Paraphimosis,) which usually occurs in young persons, because of the small preputial opening, and which frequently causes much anxiety to parent and patient, as well as trouble to the medical attendant.

The œdema which takes place in the glands, as well as in the loose tissue above the stricture, can, by well applied pressure with the hand or fingers brought to a cone, be readily overcome in a few minutes, and with little or no pain to the patient, and then by a sudden and dexterous movement, the everted prepuce can be brought forward over the now much reduced glands, and your patient may now be up and "go his way rejoicing," whilst in the plan of reducing the trouble by cold applications, astringents, etc., or later, by the knife and scissors, much delay is brought about, and frequently much pain endured.

I am prompted to state this simple plan because of the fact that I have never seen mention of it in print, and with one exception have never known it tried by any member of the profession in this section of country, and if this simple statement prove the means of relieving but one unfortunate little fellow, (without the knife,) I shall be amply repaid for this scribble.

---

*Citric Acid in the Treatment of Cancer.*—In *The British Medical Journal*, of November 27th, John H. Woods, M. D., reports a case, the cancer of the œsophagus and cardiac orifice of the stomach, in which the symptoms were, for a time, very much relieved by the use of citric acid in large doses, combining it, on Dr. Sidney Ringer's plan, with wine of ipecac in minim doses.

